# Handling persistent cardiac troponin elevation in asymptomatic athletes: The role of macrocomplexes

**DOI:** 10.1016/j.ahjo.2026.100804

**Published:** 2026-05-25

**Authors:** Simon Wernhart, Martin Halle, Sebastian Zenk

**Affiliations:** a Department for Preventive Sports Medicine and Sports Cardiology, TUM School of Medicine and Health, TUM University Hospital, Technical University of Munich (TUM), Munich, Germany; b DZHK (German Centre for Cardiovascular Research), partner site Munich Heart Alliance, Munich, Germany; c Synlab MVZ Augsburg, Germany

**Keywords:** Myocarditis, Athlete, Troponin I, Macro troponin I, troponin T, return-to-play

## Abstract

**Study objective:**

Return-to-play (RTP) decision includes cardiac troponin I returning to baseline. Formation of antibodies to cardiac troponin I (macrocomplexes) may lead to persistent, false positively elevated cardiac troponin I and may induce unnecessary exclusion from competitive sports.

**Design:**

Single-centre retrospective study.

**Setting:**

Sports cardiological outpatient clinic.

**Participants:**

We investigated 12 asymptomatic athletes who had clinically recovered from their first episode of acute myocarditis, but who showed persistently elevated cardiac troponin I levels without kinetics.

**Interventions:**

Testing for the presence of macrocomplexes using an Abbott high-sensitivity cardiac troponin I assay with and without polyethylene glycol (PEG) precipitation to precipitate any potential macrocomplexes present in the samples.

**Main outcome measures:**

Prevalence of a cardiac troponin I recovery rate in PEG precipitation.

**Results:**

13 samples from 12 athletes (mean age 36.2 ± 12.3 years) were examined with a cardiac troponin I range from 35.4 ng/L to 951 ng/L. After PEG precipitation, a measurable cardiac troponin I concentration was detected in only two cases. In 12 of the 13 samples examined, the cardiac troponin I recovery rate was below 35%, which suggests the presence of macrocomplexes. Mean time from diagnosis of acute myocarditis to assessment of macrocomplexes was 6.2 ± 3.6 months.

**Conclusion:**

Formation of macrocomplexes seems to be highly prevalent in athletes who have clinically recovered from acute myocarditis. PEG precipitation may be a feasible laboratory approach to help in the clinical decision-making of athletes to avoid unnecessary exclusion from competitive sports.

## Highlights

• Persistent and stable elevation of cardiac troponin I can be the result of macrocomplex formation.
• Return-to-sports following unremarkable sports cardiological examination and presence of macrocomplexes may be feasible.
• Polyethylene glycol precipitation could be a feasible approach to assess macrocomplexes in sports cardiological routine.

## 1 Introduction

Cardiac troponin has become a sensitive marker to detect cardiac injury, either in the form of acute inflammation of the peri-myocardium (inflammatory myopericardial syndrome) [1] or acute coronary syndrome [2]. Return-to-play (RTP) following acute myocarditis usually requires a clinically asymptomatic individual, insignificant electro- and echocardiography, exertional exercise testing without induction of arrhythmias, and normalization of cardiac biomarkers [3], [4]. Depending on the extent of cardiac involvement and morphological residuals (such as late gadolinium enhancement, LGE) bearing arrhythmogenic potential, RTP is usually achieved between three to six months following diagnosis [5]. Recent reports propagate individualized and potentially earlier clearance for RTP in the absence of red flag symptoms (exertional chest pain, palpitations, unexplained dyspnea or syncope), left ventricular dysfunction or complex arrhythmias, suggesting a more liberal approach to a stepwise RTP starting four weeks following diagnosis [6].

Assessment of cardiac biomarkers beyond the acute phase of perimyocarditis is frequently performed if unspecific symptoms, such as palpitations or persistent exercise limitations, are described by athletes striving to regain their former level of performance. Sometimes cardiac troponin may even be analyzed in the absence of symptoms. Persistent elevation of cardiac troponin in an otherwise asymptomatic athlete can cause diagnostic uncertainty, may induce unnecessary and expensive investigations and may impose a psychological burden on the athlete.

Persistence of cardiac troponin I elevation following myocarditis can be observed in up to 50% of individuals due to formation of (cellular or humoral) antibodies competing with assay-specific antibodies [7]. This may trigger persistently elevated cardiac troponin I in a completely recovered athlete who should clinically be cleared for RTP. Thus, unnecessary laboratory diagnostics and delayed RTP may follow.

We aimed to analyze the presence of macrocomplexes in athletes following insignificant RTS examination but persistently elevated cardiac troponin I without significant kinetics.

## 2 Materials and methods

### 2.1 Study population

We investigated 12 asymptomatic athletes who had clinically recovered from a first episode of acute myocarditis and had undergone insignificant sports cardiological assessments including electrocardiography, resting echocardiography and exercise testing. These athletes had received repetitive testing of elevated cardiac troponin I without clinically relevant dynamics. This was followed by an assessment of cardiac troponin T, which had been negative, but decision to RTP remained equivocal. Therefore, additional blood samples were obtained following at least 24 h of reported sedentary behavior to assess for macrocomplexes. Approval was obtained from the local ethics committee (2025-604-S-SB).

### 2.2 Laboratory approach

Testing for the presence of macrocomplexes in the blood of the study population was performed using an Abbott high-sensitivity cardiac troponin I assay with and without polyethylene glycol (PEG) precipitation as described in the manufacturer's instructions Based on the results, a cardiac troponin I recovery was calculated. (Abbott package insert, G92641R03, Oct. 2020). For the high-sensitivity cardiac troponin I assay, a limit of quantification of 5.1 ng/L was used according to the Abbott kit instructions. Abnormal (elevated) test results were defined as troponin values above the 99th percentile.

For cardiac troponin I results ≥26.2 ng/L, PEG precipitation was performed to precipitate any potential macrocomplexes present in the sample. Macrocomplexes are formed by binding of endogenous antibodies to troponin. These antigen-antibody complexes can cause elevated cardiac troponin measurements [8].

For PEG precipitation, the patient serum was diluted 1:1 with a 25% PEG 6000 solution (at least 180 μL serum +180 μL 25% PEG solution). The mixture was vortexed for 10 s and then centrifuged (5 min, 3300 *g*). Cardiac troponin I (after PEG precipitation) was then measured again from the supernatant.

Cardiac troponin recovery was calculated using the formula: Recovery = {[cardiac troponin I] (after PEG-precipitation)) * 2 / [cardiac troponin I] (before PEG-precipitation)} * 100. A recovery of ≤35% was supposed to indicate an analytical interference, i.e., the presence of macrocomplexes [8]).

We aimed to assess the prevalence of a cardiac troponin I recovery rate of ≤35% suggestive of the presence of macrocomplexes in a selected cohort of athletes with persistently elevated cardiac troponin I without kinetics and negative cardiac troponin T despite clinical recovery. Cardiac troponin T was obtained from the patients' medical history and was not measured in our laboratory.

## 3 Results

### 3.1 Patient characteristics

We included 12 athletes (nine males, three females, mean age 36.2 ± 12.3 years) from our outpatient clinic with a history of myocarditis. All raw data on patient characteristics, as well as the results of the troponin I measurements before and after PEG precipitation, can be found in Table 1. The diagnosis had been initially confirmed by magnetic resonance imaging showing myocardial oedema but no LGE. For evaluation of RTP, cardiac troponin I levels were assessed, which revealed increased cardiac troponin *I* ≥ 26.2 ng/L. Additional analysis of cardiac troponin T levels revealed normal results for all individuals. One of the athletes was investigated twice. All individuals displayed preserved biventricular ejection fraction without wall motion abnormalities. In ten patients, follow-up imaging was performed between four weeks and six months showing resolution of oedema; LGE did not develop in any athlete. Mean time from diagnosis of acute myocarditis to assessment of macrocomplexes was 6.2 ± 3.6 months.

**Table 1:** Patient characteristics, troponin I raw data and calculated recovery rate.

Patient	Characteristics		Cut-off 35%				Cut-off 20%		
Age [years]	Gender	Fig. 1: Sample ID	A Before PEG Cardiac Troponin I [ng/L]	After PEG Cardiac Troponin I [ng/L]	B Recovery [%]	Fig. 1 Sample ID	C Before PEG Cardiac Troponin I [ng/L]	After PEG Cardiac Troponin I [ng/L]	D Recovery [%]
28	m	1	**37.4**	**<5.1**	**27.3**	[1]	*[37.4]*	*[<5.1]*	*<20%*
30	m	2	**204.3**	**18.9**	**18.5**	2	**204.3**	**18.9**	**18.5**
57	m	3	**47**	**<5.1**	**21.7**	[3]	*[47]*	*[<5.1]*	*>20%*
20	m	4	**39.1**	**<5.1**	**26.1**	[4]	*[39.1]*	*[<5.1]*	*>20%*
43	m	5	**62.3**	**<5.1**	**16.4**	5	**62.3**	**<5.1**	**16.4**
49	m	6	**951.9**	**76.5**	**16.1**	6	**951.9**	**76.5**	**16.1**
36	f	7	**39**	**<5.1**	**26.2**	[7]	*[39]*	*[<5.1]*	*>20%*
33	m	[8]	*[26.7]*	*[<5.1]*	*>35%*	[8]	*[26.7]*	*[<5.1]*	*>20%*
33	m	9*(2025/07)	**63.8**	**<5.1**	**16.0**	9*(2025/07)	**63.8**	**<5.1**	**16.0**
32	m	10	**35.4**	**<5.1**	**28.8**	[10]	*[35.4]*	*[<5.1]*	*>20%*
	m	11*(2025/08)	**170.7**	**<5.1**	**6.0**	11*(2025/08	**170.7**	**<5.1**	**6.0**
47	f	12	**111.3**	**<5.1**	**9.2**	12	**111.3**	**<5.1**	**9.2**
24	f	13	**79.3**	**<5.1**	**0.1**	13	**79.3**	**<5.1**	**0.1**
**Average m**		**MIN**	35.4	–	0.1	**MIN**	62.3	–	0.1
**36** ± **10.8 years**		**MAX**	951.9	–	28.8	**MAX**	951.9	–	18.5
**Average f**		**MEAN**	153.5	–	17.7	**MEAN**	234.8	–	11.7
**35.7** ± **9.4 years**		**MEDIAN**	63.05	–	17.4	**MEDIAN**	111.3	–	16.0
**Study samples**	**13**	**Study samples**	**12**	**12**	**12**	**Study samples**	**7**	**7**	**7**

The table contains the underlying raw data from our study. All excluded patient samples are indicated in square brackets, and the corresponding data are shown in italics. The calculations are presented in the lower rows. MIN: minimum measured or calculated value. MAX: maximum measured or calculated value. MEDIAN: calculated median- MEAN: calculated mean. Study samples: number of included samples in the respective analysis. Samples 9 and 11 originate from the same patient (marked with an asterisk) but were collected at different time points, as indicated. In both cases, macrocomplexes were detected. Excluded samples are depiced in square brackets. m: male, f: female.

The level of significance was set at alpha <.05.

### 3.2 Cardiac troponin I recovery

**Fig. 1:**
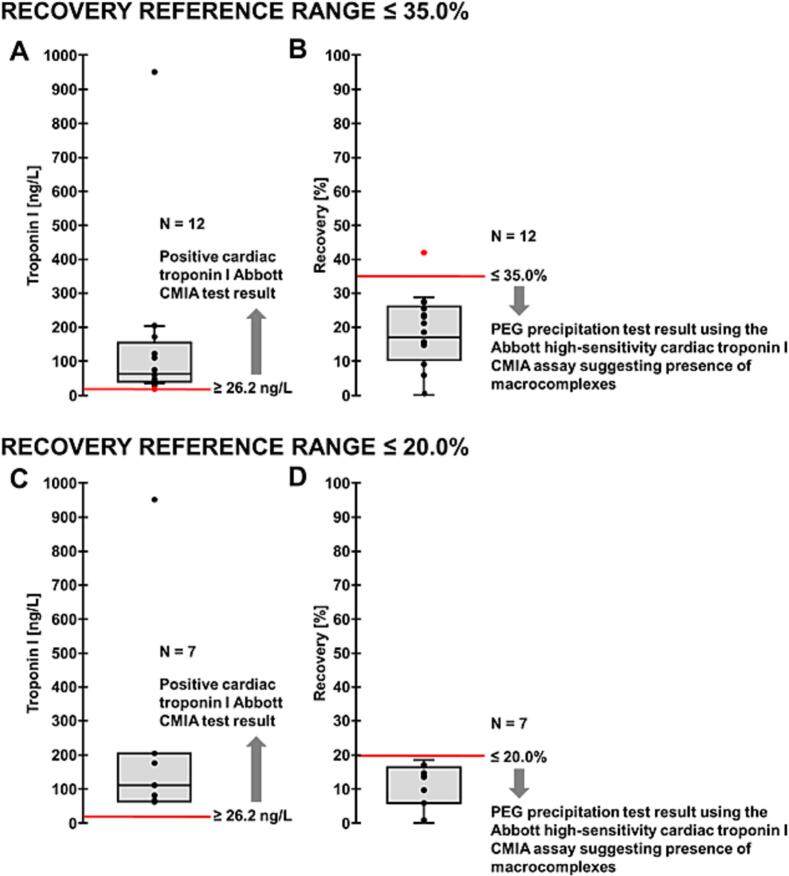
Cardiac troponin I measurement and recovery. Boxplots for measurement of cardiac troponin I (A) and recovery rates (B) in 12 asymptomatic athletes clinically recovered from myocarditis. (A) Measured cardiac troponin I. Results from one patient (red dot) with an initial cardiac troponin I concentration of 26.7 ng/L and a calculated recovery rate of ≤38.2% were excluded. Positive results for cardiac troponin I represent values of ≥26.2 ng/L in a chemiluminescent microparticle immunoassay (CMIA) according to the manufacturer's specifications. Initially measured cardiac troponin I concentrations are displayed. (B) Recovery rates for cardiac troponin I. A recovery rate ≤ 35% in polyethylene glycol (PEG) precipitation using the Abbott high-sensitivity cardiac troponin I assay may suggest the presence of macrocomplexes according to the current literature [8]. Figure (C) and (D): In addition to the 35% cut-off, the data were also evaluated using a 20% cut-off for the detection of troponin macrocomplexes, analogous to the analyses shown in Figures (A) and (B). (For interpretation of the references to colour in this figure legend, the reader is referred to the web version of this article.)

Initially, the subjects showed cardiac troponin I concentrations ranging from 35.4 ng/L to 951 ng/L (mean 153.5 ± 257.4 ng/L, Fig. 1A). After PEG precipitation, a measurable cardiac troponin I concentration was detected in only two cases.

In all other cases, cardiac troponin I was below the assay's detection limit (< 5.1 ng/L). Thus, for 12 of the 13 samples examined, the cardiac troponin I recovery rate was ≤35% (Fig. 1B). Due to technical limitations (limited assay linearity), the excluded sample resulted in a recovery rate of ≤38.2%. Compared with the other samples, the cardiac troponin I level here was only marginally elevated at 26.7 ng/L. In addition to the 35% cut-off, the data were also evaluated using a 20% cut-off for the detection of troponin macrocomplexes (Fig. 1C and D).

## 4 Conclusions

We show that in asymptomatic post myocarditis athletes with persistently and stable elevated cardiac troponin I and normal sports cardiological RTP assessment, PEG analysis provides support for false positive laboratory results due to the presence of macrocomplexes as a laboratory phenomenon. To our knowledge, there is currently no evidence that the presence of macrocomplexes in athletes has an impact on the incidence of adverse cardiac events, such as malignant arrhythmia, cardiovascular events or mortality. A strength of our study was that the diagnosis of myocarditis was not only based on clinical, electrocardiographical, and biomarker data, but was given following current guidelines using magnetic resonance imaging as the gold standard to detect acute inflammation on T2-weighted imaging [9].

It has been reported that acute exercise can induce cardiac troponin I elevations above the 99th percentile after 30 to 55 km of walking, which independently predicted more cardiovascular events and higher mortality in a cohort of older long-distance walkers [10]. This has been attributed to myocardial stress and short episodes of local ischemia, increased membrane permeability of the sarcolemma of cardiomyocytes and even increased apoptosis or accelerated cardiomyocyte turnover [11]. However, data on prolonged or chronic cardiac troponin I elevation in athletes is lacking.

Assessment of cardiac troponin I following myocarditis is sometimes added even in a clinically asymptomatic athlete with normal sports cardiological assessment, consisting of electrocardiography, echocardiography and exertional exercise testing. In case of positive findings, cardiologists should be aware of the formation of macrocomplexes and should be educated on potential pitfalls regarding cardiac troponin assessment, including macrocomplex formation during or after cardiac disease (including perimyocarditis, acute coronary syndrome or structural heart disease). Moreover, the impact of recent bouts of exercise on cardiac troponin values in the absence of underlying cardiac disease should also be considered. This is of particular importance to avoid unnecessary withdrawal from competitive sports or non-eligibility assessment. These findings should then not lead to additional testing or even invasive diagnostic investigations.

It has been shown that cardiac troponin release differs between troponin I and T in high-performance athletes, suggesting that if cardiac troponin is analyzed in athletes, troponin T might be better suited compared to troponin I to separate physiological response from cardiac injury [12]. However, it has been suggested that troponin T may not only be present in cardiac disease but is released during strenuous exercise as a result of skeletal muscle work and injury: It has been shown, for instance, that troponin is released in exercising carriers of Duchenne muscular dystrophy without cardiac involvement, suggesting skeletal muscle involvement or laboratory interference [13], [14]. Thus, differentiation between cardiac and skeletal muscle involvement may be challenging in specific diseases [13], [15]. Increases of high-sensitive troponin T were observed in athletes between 0.7 through 25 h following a 2.5-h bout of running, with a peak at 2.9 h after the midpoint of the running episode [16]. Apart from exercise duration, sports-specific kinetics of troponin T release were observed, with rowing leading to higher release than other sports [16]. This is not surprising, as this sport involves a larger number of upper and lower body muscles at a considerable intensity of exercise. Marathon running seems to result in immediate release of peak troponin T values with normalization within 72 h following the race [17]. Thus, sports cardiologists need to consider type of exercise, duration, intensity and age when interpreting troponin kinetics [18].

A recent study has shown that when a cut-off residual activity after PEG precipitation of ≤20% was used, the diagnostic performance of PEG precipitation using Protein A immunoglobulin depletion had a high specificity of 92% using the Siemens hs-cTnI Vista assay (*n* = 189) and 95% specificity using the Abbott hs-cTnI Architect assay (*n* = 242) [19]. This could support PEG precipitation as a cost-effective way to assess macrocomplexes without further need for more complex and expensive laboratory testing.

While macrocomplex formation has been described as a common laboratory phenomenon [7], its prognostic role in athletes remains unknown. Currently, there is no clear evidence that athletes with macrocomplexes are exposed to a higher risk of adverse events when exercise is resumed. Assessment of macrocomplexes could be an additional tool in clinical decision-making to allow stepwise RTP after myocarditis in athletes with unspecific symptoms, such as reduced exercise capacity or palpitations without a clear substrate in magnetic resonance imaging, Holter monitoring or exercise testing. While awareness of the laboratory phenomenon of macrocomplex formation needs to be sharpened in cardiologists assessing athletes after myocarditis, its clinical and prognostic value still needs to be determined.

### 4.1 Limitations and considerations

Although PEG testing, in addition to clinical assessment, can provide further support for clearance of athletes, the method has not been sufficiently validated against a cohort of patients with acute coronary syndrome to define cut-off values for the presence of macro troponin.

For this reason, the data presented here are purely experimental. Cut-off values for the recovery rate were taken from the literature [8], with values ≤35% indicating an analytical interference and thus suggesting the presence of macrotroponin. Due to the lack of validation, there is currently no appropriate control available for the experimental macrotroponin test. However, this test was performed together with the PEG precipitation for prolactin that is well established in the laboratory. Similar to troponin, prolactin is also measured using an immunoassay. In the case of prolactin, the aim is to rule out macroprolactin as the cause of hyperprolactinemia. The same testing scheme was applied for both (prolactin and troponin) PEG precipitations. However, in contrast to the troponin precipitation, different cut-off values were used for the prolactin precipitation. A recovery rate > 60% indicates the presence of predominantly monomeric prolactin without macroprolactin. A recovery rate below 40% suggests that the sample contains predominantly macro prolactin or oligomeric prolactin. A grey zone is also defined for recoveries between 40% and 60% [20]. Although the thresholds for macrotroponin (≤ 35%) and macroprolactin (≤ 40%) are similar, it is possible that adjustments will be necessary in the future. One possible reason for this could be that PEG precipitation can also produce nonspecific results. In this context, a recent study shows that when a stricter cut-off is applied (recovery ≤20%), a specificity of approximately 92–95% is achieved [19].

In addition to the PEG precipitation used here, other methods for detecting macrotroponin are also available. In a case report, gel filtration and protein A IgG precipitation were able to identify an immunoreactive high-molecular-weight protein (macrotroponin) [21]. In a similar approach combined with immunoassays using a NAb™ Protein A spin column technique, a recovery rate of 40% was achieved, which was considered to be based on the presence of macrotroponin [22], [23].

In contrast to the PEG precipitation used in our approach, the costs for a column-based purification are significantly higher and are not financially feasible for routine clinical use. Therefore, feasibility and cost considerations in routine clinical practice must also be regarded as potential limitations.

Due to the lower limit of linearity of the assay used here (5.1 ng/L) and the underlying mathematical constraints, a PEG precipitation for macrotroponin in this experimental setting is only meaningful for samples with a cardiac troponin I concentration greater than 29.2 ng/L when the recovery rate is ≤35%. In particular, for the sample excluded from this study (troponin I 26.7 ng/L, calculated recovery rate ≤ 38.2%), a precise calculation was not feasible due to this limitation.

The conclusions rely exclusively on PEG precipitation. However, PEG is a non-specific screening tool [24]. It does not definitively confirm the presence of macrocomplexes nor does it allow identification of the type of interference (troponin I or T). Confirmatory techniques (e.g., immunoglobulin depletion, chromatography) would be necessary to demonstrate the presence of macrocomplexes of troponin I. Larger studies using different approaches to analyze circulating macrocomplexes, such as chromotagraphic or Western blot analyses [25], [26], [27], [28], are necessary to confirm our results in a larger sample size. It has been recently suggested that the analysis of different molecular circulating forms of cardiac troponins could be very useful to separate benign cardiac troponin T elevations after strenuous exercise from those of acute myocardial infarction [29]. In addition, novel immunoassays specific for the long forms of cardiac troponins have been developed [29], [30]. Whether specific assays of long circulating forms of cardiac troponins could be a more specific biomarker of acute myocardial infarction or acute myocarditis in athletes compared to total high-sensitive cardiac troponin I or T assays, needs to be determined [31], [32].

The International Federation of Clinical Chemistry and Laboratory Medicine (IFCC) has provided useful recommendations for laboratories to overcome analytical obstacles and avoid false-positive troponin measurements [24]. Knowledge of such recommendations is important for clinicians and laboratory experts alike. PEG precipitation protocols advocate non-specific precipitation of most proteins rather than those of a target single-antibody immune complex. Consequently, PEG precipitation does not address the origin of the patient's cardiac troponin assay interference. Furthermore, different cardiac troponin assays are affected by the presence of PEG in the sample to different degrees [24], which can confound interpretation and may result in incorrect clinical decisions and unjustified withdrawal of athletes from sports competitions. IFCC recommendations suggest a stepwise approach to exclude interference: First, repeated testing with the initial cardiac troponin assay following re-centrifugation, dilution, or with an alternate specimen type (plasma or serum) should be done [24]. As a second step, an alternative cardiac troponin assay is recommended, followed by other methods, such as PEG precipitation. The IFCC document states that discordant results should raise suspicion of interference if the increase in cardiac troponin remains stable over time with low clinical pre-test probability of underlying disease, or if alternative tests yield results below the upper reference limit. If cardiac troponin concentrations differ between two tests by more than 3–5-fold (in the absence of myocardial infarction), interference is most likely present and seems to occur in up to 50% of cases [24]. While PEG precipitation is cheap and broadly available, it may miss cardiac troponin-assay interference and different cardiac troponin assays are affected differently by presence of PEG in the sample. It is not known, what exactly is precipitated in the sample. If suspicion of interference remains, gel filtration or sucrose ultracentrifugation can be of additional value [24]. Establishing two different cardiac troponin assays may help reduce the risk of false positive [24], but this was not available in our laboratory due to local policy. Our data and experience will induce collaboration with other laboratories applying different assays. Not only laboratories, but also clinicians need to apply a high degree of suspicion for interference in the absence of high clinical likelihood of cardiac disease. Efforts should be made to promote broader collaborations between different laboratories (using different assays) and clinics to prevent unnecessary additional diagnostics. Noteworthy, the frequency of macrotroponin vary by assay, and some macrotroponins can be inhibitory. Such caveats have been published and must be considered when the strategy of laboratory-specific interference is discussed [8], [24], [31], [32].

Our cohort showed stable elevation of cardiac troponin I, which is suggestive of analytical interference behind the background of a low clinical likelihood of undetected underlying disease. On the other hand, a changing pattern of values makes the likelihood of an analytic confounder far less likely. As the literature on macrotroponin is extensive [8], [24], [31], [32], [33], [34], but clinical awareness is insufficient in our region, we will adapt a closer collaboration and interdisciplinary discussion of cases in which clinical and laboratory findings are contradictory. Likewise, availability of two assays for cardiac troponin is essential to prevent unnecessary testing.

Our study population is highly selected. Only athletes in whom cardiac troponin I was clinically measured and found persistently elevated were included. This introduces significant selection bias and precludes any inference regarding the prevalence of analytical interferences in this setting. The absence of a control group further limits interpretability and is a major limitation: Future studies will be necessary to analyze the formation of macrocomplexes in an athlete control group during the acute and subacute phases of myocarditis. Since this population is highly selective, a multicenter approach may need to be taken. However, interference can also be expected in non-athletes after myocarditis and should be considered by both laboratory experts and clinicians. In addition, studies have described the formation of macrocomplexes because of COVID-vaccination [33], [35]. However, in our study 11 athletes had received prior COVID-vaccination, which was not temporally related to the event of acute myocarditis. However, long-term persistence of macrocomplexes because of COVID-vaccination cannot be entirely excluded.

Although magnetic resonance imaging did not reveal signs of structural heart disease in any athlete, it should be considered that subclinical heart disease can lead to persistent troponin elevations. Also, paroxysmal asymptomatic supraventricular or ventricular arrhythmias can lead to troponin release. Holter monitoring was only performed in the acute phase of myocarditis, revealing no arrhythmias in this cohort, and our athletes did not report any symptoms of arrhythmia during follow-up. As ongoing rhythm monitoring during the follow-up period was not available, paroxysmal asymptomatic tachycardia as a reason for troponin elevation cannot be entirely excluded. In addition, subclinical re-infection during follow-up may explain troponin elevation, but was clinically unlikely in our asymptomatic athletes.

Nevertheless, we would like to emphasize that according to the Abbott kit instructions (G9264, revised Oct. 2020), falsely elevated troponin values may arise not only from immunoglobulin-related interference (e.g., macrotroponin), but also from a range of other analytical and pre-analytical factors. These include heterophile antibodies and human anti-animal antibodies, as well as rheumatoid factor–mediated assay interference. Additional causes comprise sample-related issues such as fibrin clots, hemolysis, and biotin supplementation, alongside endogenous interferences like elevated alkaline phosphatase or lipemia. In rare cases, skeletal muscle disease or assay antibody cross-reactivity may also lead to spurious troponin elevations.

In our study, many of these potential confounders could be excluded based on clinical history and laboratory assessment. From our laboratory experience, analytically relevant human anti-animal antibody interference is a rare finding and does not occur at the frequency observed in our study cohort. Furthermore, interference from biotin is not a relevant issue for the Abbott high-sensitivity cardiac troponin I assay used in our analysis.

Our study was retrospective in nature and was only performed using PEG precipitation for cardiac troponin I, while it would also be interesting to extend the study to athletes with persistently elevated cardiac troponin T without kinetics. The prevalence of macrotroponin T appears to be lower than that of troponin I [34], which may suggest cardiac troponin T as the better marker to test in athletes following myocarditis.

Noteworthy, considering the extensive literature on macrotroponin [8], [24], [31], [32], [33], [34], clinical and cardiological recommendations should be more alert of potential analytical interference and should implement IFCC recommendations [24] as a framework for optimized cardiological care in both athletes and regular patients to avoid unnecessary testing and stigmatization of patients in the absence of underlying disease.

### 4.2 Summary

In summary, we provide evidence for the feasibility of PEG precipitation to potentially distinguish underlying cardiac disease from false positively elevated cardiac troponin I levels, being macrocomplexes, in asymptomatic athletes after myocarditis. While routine cardiac troponin assessment should be avoided in insignificant RTP-examinations of athletes, PEG precipitation could assist in clinical decision-making for RTP in athletes when cardiac troponin I levels are elevated. The prognostic role of macrotroponin in athletes has yet to be determined.

## List of abbreviations

hs-cTnI: High-sensitive cardiac troponin I
LGE: Late gadolinium enhancement
PEG: Polyethylene glycol
RLU: Relative light units
RTP: Return-to-play

## Authors’ information

SW is a cardiologist with a focus on sports cardiology, cardiomyopathies and heart failure. He is also a trained sports scientist. MH is a sports cardiologist and task force member of the current European guidelines of sports cardiology. SZ is laboratory physician with an expertise in the analysis of cardiac biomarkers.

## CRediT authorship contribution statement

**Simon Wernhart:** Writing – review & editing, Writing – original draft, Project administration, Methodology, Conceptualization. **Martin Halle:** Writing – review & editing, Writing – original draft, Supervision. **Sebastian Zenk:** Writing – review & editing, Writing – original draft, Project administration, Formal analysis, Conceptualization.

## Ethics approval and consent to participate

Approval was obtained from the local ethics committee (2025-604-S-SB). Consent for anonymous publication was obtained from all patients. The study was performed in accordance with the Declaration of Helsinki.

## Funding

There was no funding to this study.

## Declaration of competing interest

SW has received honoraria for lectures from Bristol-Myers Squibb. MH reports honoraria for lectures from Abbott, Amgen, Astra- Zeneca, Boehringer-Ingelheim, BMW, Bristol-Myers Squibb, Daiichi-Sankyo, Lilly, Medi, MSD Sharp & Dohme GmbH, Norsan, Novartis, Pfizer and Roche, consulting fees from Medical Park. No other potential conflicts of interest are reported and none of the reported interests are related to this manuscript.
